# Sternal plate fixation for sternal wound reconstruction: initial experience (Retrospective study)

**DOI:** 10.1186/1749-8090-6-63

**Published:** 2011-04-29

**Authors:** Hosam Fawzy, Kannin Osei-Tutu, Lee Errett, David Latter, Daniel Bonneau, Melinda Musgrave, James Mahoney

**Affiliations:** 1 Division of Cardiovascular and Thoracic Surgery, Department of Surgery, Terrence Donnelly Heart Center, Keenan Research Center in the Li Ka Shing Knowledge Institute of St. Michael's Hospital, University of Toronto, 30 Bond Street, Toronto, Ontario, M5B 1W8, Canada; 2 Division of Plastic Surgery, Department of Surgery, Terrence Donnelly Heart Center, Keenan Research Center in the Li Ka Shing Knowledge Institute of St. Michael's Hospital, University of Toronto, 30 Bond Street, Toronto, Ontario, M5B 1W8, Canada

**Keywords:** Sternal Plating, Sternal Dehiscence

## Abstract

**Background:**

Median sternotomy infection and bony nonunion are two commonly described complications which occur in 0.4 - 5.1% of cardiac procedures. Although relatively infrequent, these complications can lead to significant morbidity and mortality. The aim of this retrospective study is to evaluate the initial experience of a transverse plate fixation system following wound complications associated with sternal dehiscence with or without infection following cardiac surgery.

**Methods:**

A retrospective chart review of 40 consecutive patients who required sternal wound reconstruction post sternotomy was performed. Soft tissue debridement with removal of all compromised tissue was performed. Sternal debridement was carried using ronguers to healthy bleeding bone. All patients underwent sternal fixation using three rib plates combined with a single manubrial plate (Titanium Sternal Fixation System^®^, Synthes). Incisions were closed in a layered fashion with the pectoral muscles being advanced to the midline. Data were expressed as mean ± SD, Median (range) or number (%). Statistical analyses were made by using Excel 2003 for Windows (Microsoft, Redmond, WA, USA).

**Results:**

There were 40 consecutive patients, 31 males and 9 females. Twenty two patients (55%) were diagnosed with sternal dehiscence alone and 18 patients (45%) with associated wound discharge. Thirty eight patients went on to heal their wounds. Two patients developed recurrent wound infection and required VAC therapy. Both were immunocompromised. Median post-op ICU stay was one day with the median hospital stay of 18 days after plating.

**Conclusion:**

Sternal plating appears to be an effective option for the treatment of sternal wound dehiscence associated with sternal instability. Long-term follow-up and further larger studies are needed to address the indications, benefits and complications of sternal plating.

## Background

The median sternotomy incision remains appealing because it offers advantages paramount to cardiac surgery. It can be performed quickly, provides excellent exposure of vital chest structures, affords the safety of central cannulation for cardiopulmonary bypass, and is well tolerated by most patients [1].

Since Julian re-introduced Milton's operation for median sternotomy in 1957 [2], numerous methods for sternal fixation have been described. The common mechanism leading to major and minor sternal complications is the inability to maintain stabilization of the sternotomy closure site. The current standard technique for sternal closure remains the cerclage stainless steel wires. This technique under normal physiologic loads can lead to inadequate fixation and sternal dehiscence [3]. This happens when mechanical stresses are concentrated at the steel wires, causing them to cut into the bone, and allowing variable degrees of motion to occur at the closure site. A sternal wound complication following cardiac procedures is a multifactorial problem, including numerous patient related variables as well as operative and postoperative factors. Bacterial contamination in the face of sternal separation and instability can then progress to deep sternal wound infection and mediastinitis. Off centre sternotomy, osteoporosis and advancing age may contribute to the inability of wire fixation to maintain stability leading to sternal nonunion. Effective rigid closure of the sternum may prevent post-sternotomy mediastinitis by affording greater stability and promoting primary healing of the sternum.

The aim of this study is to determine whether the transverse sternal plate fixation system is an effective treatment for postoperative wound complications associated with sternal dehiscence secondly to see if it improves the mechanical stability of sternal closure and to evaluate its initial outcome.

## Patients and methods

### Patients

With approval of the St. Michael's Hospital Research Ethics Board and the University of Toronto, this retrospective review study included consecutive patients who underwent sternal plating during the period from July 2004 to January 2008. Patients were considered for plate fixation after failure of the primary standard wire closure associated with wound dehiscence. Informed consent was obtained for every procedure. Follow up includes all in hospital complications and following discharge up until one year with no patients lost to follow up.

### Operative procedure: (Figure 1)

**Figure 1:**
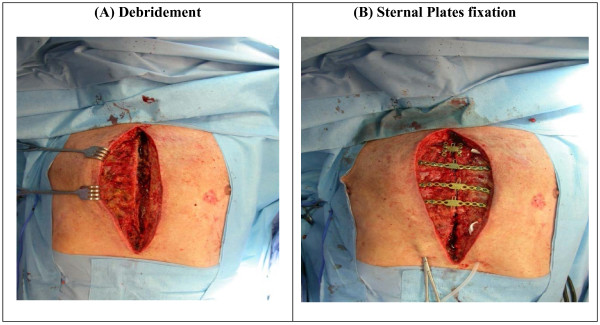
**Surgical technique of sternal plates' fixation**. It shows wound debridement and pectoral flap development followed by sternal fixation using four plates.

Under general anesthesia with endotracheal intubation, the patient was placed in the supine position with both arms tucked along the sides to avoid stretching on the pectoralis muscles and to facilitate reducing the sternal separation. Debridement was begun with excision of all wound edges, including skin, subcutaneous tissue and any necrotic tissue until they were free of the devitalized tissue and down to the bleeding tissue. Hemostasis was obtained. The old sternal wires were removed and all nonviable bone and cartilage was removed down to the level of bony cortex and marrow cavity. Bone biopsy was sent to microbiology for culture.

To expose the ribs bilaterally, pectoralis major muscles were elevated with overlying soft tissue from the midline to the level of the mid-clavicular line to create flaps and permit later approximation in the midline. The intercostals perforating vessels were divided with cautery. It was not necessary to perform a second incision at the shoulder to release the pectoral muscle insertion. Subsequently, the entire wound was lavaged with 3 liters of warm normal saline. The two sternal halves were brought together using two large reduction forceps on both the superior and inferior aspects of the sternum.

Four titanium plates (Synthes Titanium Sternal Fixation System, Synthes CMF, West Chester, PA, USA) were placed transversely across the two sternal halves at the level of the second, third, fourth ribs, and one manubrial plate in most circumstances. Using a template, the plates were contoured. The appropriate length of the plate was selected to allow a minimum of four locking screws on each side. The holes were drilled in bone and cartilage with the aid of the drill guide to precisely create the drill hole depth avoiding injury to the underlying structures. Depth was assessed at times with the depth gauge but recently by analysis of sternal and rib thickness by CT scan. Using the measurement tool sternal and rib thickness can be assessed preoperatively. It was also important to avoid the inferior margin of the rib to avoid injury to the intercostals vessels and nerves. Screw lengths varied among patients and ranged from 12 to 18 mm in length, with 12 mm being the most commonly used size. Once the plates were secured in place, the reduction forceps were removed.

**Figure 2:**
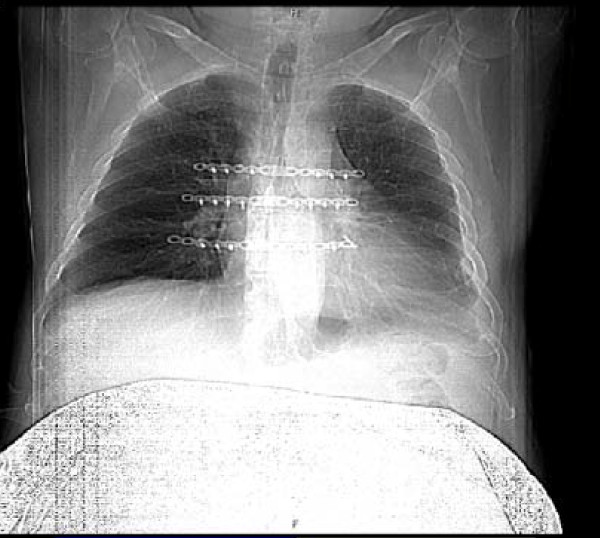
**Post-operative Chest X-ray following sternal plates' fixation**. It demonstrates sternal union.

Two Jackson-Pratt no. 10 drains were placed one under each muscle flap, through two separate small incisions along the lower edge of the sternotomy wound. The muscles were approximated at the mid line with interrupted no. 1 Vicryl sutures. Superficial muscle fascia and subcutaneous tissues were closed with 2-0 Vicryl sutures. The skin was closed with staples. A postoperative chest radiograph was obtained routinely in every patient (figure 2) to confirm the position of the plates and exclude pneumothorax.

Sternal precautions were observed for the first 6 week postoperatively with, avoidance of activity that would place stress on the pectoral region. Drains were removed when output was consistently less than 25 cc per drain per day. An antibiotic course for 4-6 weeks was completed post-operatively under the direction of the infectious disease service.

### Statistical Analysis

All pre-operative patients' demographic data together with operative characteristics and post-operative data were imported into Excel worksheets, for organizational purposes. Data were expressed as mean ± SD, Median (range) or number (%) using Excel 2003 for Windows (Microsoft, Redmond, WA, USA).

## Results

During the two years of the study, 40 patients underwent sternal plating. There were 31 males and 9 females. The average age was 69.7 ± 9.4 years with BMI of 47.1 ± 4.8 Kg/m^2^. Fourteen patients (35%) were diabetics, 27 (67.5%) hypertensive, 10(25%) smokers, 8 (20%) had COPD and 11 (27.5%) had renal failure. A summary of patients' characteristics is shown in Table 1.

**Table 1:** Pre-operative Patients' Demographics

Item	Patients
Age (y)	69.7 ± 9.4

Male/Female	31/9

Weight (Kg)	77 ± 9.5

BMI (Kg/m^2^)	47.1 ± 4.8

Obesity (BMI > 30 Kg/m^2^)	27 (67.5%)

Hypertension	27 (67.5%)

Diabetes	14 (35%)

Smoking	10 (25%)

Hypothyroidism	2 (5%)

Chronic Renal Failure	11 (27.5%)

COPD	8 (20%)

PVD	1 (2.5%)

Corticosteroid use	1 (2.5%)

CHF	10 (25%)

Re-operation	3 (7.5%)

Values are means ± SD or Number (%) where shown.

BMI: Body Mass Index.

COPD: Chronic Obstructive Pulmonary Disease.

CHF: Congestive Heart Failure.

PVD: Peripheral Vascular Disease.

Most often following CABG (70%), AVR (12.5%), Mitral valve repair (5%) or combined procedures (12.5%). LIMA was used in all CABG patients but 2. The average time from the heart procedure to sternal plating was 9 days (range 4-420 days). Primary operative characteristics of the patients are shown in Table 2. The decision to proceed with plating was made based on clinical assessment of the sternum or gross infection in the operating room. All patients were presented by pain and wound dehiscence. Twenty two patients (55%) were presented by sternal instability alone while 18 patients (45%) with associated wound discharge. All patients had gross instability at the time of plating. All patients had cultures from the wound at the time of surgery. The most common pathogens were coagulase -negative staphylococci (35%) and Staphylococcus aureus (17.5%). Operative cultures of eight patients (20%) showed no growth. Table 3 summarizes the different organisms detected.

**Table 2:** Primary operative characteristics of the Patients

Operation	Patients
CABG	28 (70%)

AVR	5 (12.5%)

CABG + AVR	4 (10%)

CABG + MVR	1 (2.5%)

MV Repair	2 (5%)

Total No. of grafts	3.4 ± 0.5

Use of LIMA/RIMA	26 (65%)

TBT (min)	86.9 ± 43.5

OR Time (min)	238.8 ± 58.7

Re-operation for bleeding	7 (17.5%)

Pts. required massive blood transfusion	1 (2.5%)

Values are means ± SD or Number (%) where shown.

AVR: Aortic Valve Replacement.

CABG: Coronary Artery Bypass Grafting.

LIMA: Left Internal Mammary Artery.

MVR: Mitral Valve Replacement.

Min: minutes.

OR Time: Operative Time.

RIMA: Right Internal Mammary Artery.

TBT: Total Bypass Time.

**Table 3:** Organisms detected

Organisms	Patients
Coagulase-negative Staph.	14 (35%)

Staph. Aureus`	7 (17.5%)

Gram-negative rods	5 (12.5%)

Enterococcus	2 (5%)

Coliform Bacilli	3 (7.5%)

Diphteroid Bacilli	1 (2.5%)

No Pathogens identified	8 (20%)

Values are Number (%) where shown.

In addition to surgical treatment, intravenous antibiotics were administrated for the infection, most commonly Cloxacillin (60%), followed by Vancomycin (20%). Mean duration of intravenous antibiotic treatment was 28 days following surgery, followed by oral antibiotics.

Mean operative time was 122.5 minutes. All patients healed. Postoperative wound complications included: one patient (2.5%) has post-operative bleeding. One patient (2.5%) developed postoperative seroma after 16 days. Six patients (15%) developed post-operative superficial wound dehiscence with discharge. They all subsequently healed. Four patients (10%) developed postoperative pleural effusion that was successfully drained by thoracocentesis. One patient (2.5%) developed post-operative pneumothorax that was drained by an intercostal chest tube. Two patients (5%) developed recurrent wound infection and healed with negative pressure wound therapy. Both were immunocompromised. Post-operative complications are shown in Table 4.

**Table 4:** Post-operative Complications

Complications	Patients
Post-op bleeding	1 (2.5%)

Seroma	1 (2.5%)

Superficial Wound Dehiscence	6 (15%)

Recurrent infection	2 (5%)

Hard-ware removal	2 (5%)

Pleural effusion	4 (10%)

Pneumothorax	1 (2.5%)

Values are Number (%) where shown.

**Figure 3:**
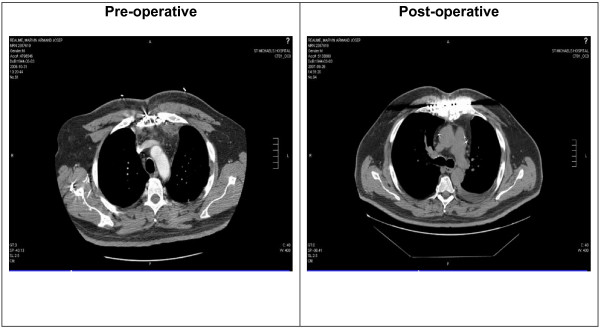
**Pre and Post-operative chest CT scan following sternal plates' fixation**. It demonstrates resolving of mediastinitis and sternal union.

Our first few patients were kept sedated and ventilated for 48 hours before extubation was attempted to minimize excessive movements that might affect the wound. Later, we have modified our post-operative care protocol, so all patients were extubated immediately after surgery unless haemodynamically unstable. Seventy five percent of the patients were extubated immediately after surgery, 10% extubated in the first 24 hours and 15% were late (> 24 hours). Those of late extubation compromised a group of six patients with severe COPD that were on the ventilator for a long time and who ultimately required tracheostomy. Median post-op ICU stay was one day (range 1-29 days). Total median hospital stay was 18 days, with a range from 3-88 days after sternal plating. The wide variability was mostly due to prolonged stay of few patients due to other medical problems not related to the sternum. There was one death unrelated to the sternal closure that had infective endocarditits of his prosthetic valve and died of refractory septic shock. Post-operative hospital course is shown in Table 5. The median follow-up time at one year revealed thoracic stability in all patients (figure 3). No patient showed clinically significant restrictive pulmonary compromise, although formal postoperative pulmonary function measurements were not obtained. Postoperative chest pain disappeared in the majority of the patients. Chronic postoperative pain was reported in two patients, the first one was well managed with oral nonsteroidal medications. The second patient required plate removal to relieve his pain.

**Table 5:** Post-operative Hospital Course

Item	Patients
Ventilation time > 24 Hrs.	6 (15%)

ICU stay (days)	1 (1-29)

Hospital stay (days)	18 (3-88)

Hospital Mortality	1 (2.5%)

Values are Number (%) or Median (range) where shown.

### Comment

Since the introduction of median sternotomy as an approach to perform open heart operations, it remains the preferred approach allowing better exposure and easy access of the heart and mediastinal structures. Two well-described complications with this type of incision are sternal dehiscence and wound infection [4-6]. Despite the relative infrequency of these complications (0.4% - 5.1%), they carry high morbidity and mortality.

Many techniques have been developed to break the vicious circle of sternal stability and infection. Continuous antibiotic irrigation was first used by Shumacker and Mandelbanum [6]. Debridement with removal of all infected tissue is emphasized no matter what the reconstructive technique is utilized. Negative pressure wound therapy has also been utilized both as a treatment for wound dehiscence and as a temporary measure to help treat the infection before sternal reconstruction. Soft tissue reconstruction has emphasized obliteration of dead space with health well vascularized tissue initially using pectoralis major flaps [4]. Subsequently, many techniques have been used that include advancement flaps, rotational flaps, and turnover flaps [7-10]. Large sternal defects associated with partial and total sternectomy have also been covered using the omentum, rectus muscle and latissimus muscle flap [11-14]. Muscle flaps can be used alone or in combination with sternal rewiring. Robicszek parasternal weave is still the standard technique used for sternal rewiring in many centers [15]. As a supplement to sternal wires, longitudinal plates had been used to fix the sternum together with circumferential wire [16-18]. Other techniques have included the X-shaped and box-shaped plates over the sternum with two figures of 8 wires placed around the manubrium and the xiphisternal junction [18].

Chest wall defects seen after complete or partial sternectomy can result in paradoxical chest wall movement and thoracic instability that is difficult to address by muscle flaps alone. Restoration of sternal or chest wall stability can be achieved with transverse locking plate fixation system by distributing the force laterally over the ribs on both sides. It relies on rib and sternal fixation when available to provide chest wall stability. Using the described technique, plate stabilization is achieved on the anterior surface of the ribs and no dissection is necessary at the deep aspect of the sternum, avoiding the risk of injury to the underlying heart structures. It is also feasible in such cases with sternal loss with little residual sternum left for fixation. However, it is not without risks or complications. Our postoperative complication rate is low particularly involving seroma as seen with other authors. We only have one patient (2.5%) in our series that developed post-op seroma requiring treatment. Cicilioni et al [19] reported higher rate of seroma formation in 5 patients (10%) in their series of 50 consecutive sternal wound reconstructions using transverse plate fixation. Hugo et al. [7] used pectoralis muscle flaps only and reported the highest rate of seroma formation (24%) in their series of 74 patients. It is obvious that seroma formation is directly related to extensive pectoral muscle dissection rather than the presence of the metal plates. This creates a dead space underneath the muscles where seroma can form. Our low rate of seroma might be related to limited pectoral muscle dissection and routine placement of two sub pectoral Jackson -Pratt drains. We left the drains in situ until the daily drainage is less 25 ml before removal. In addition we have used a technique where the deep pectoral fixation sutures are fixed to the plate, helping to obliterate dead space. Drilling too deep or using long screws carry the risk of injury to the internal mammary artery and vein and the intercostal vessels as well as the lungs and mediastinal structures. We avoid this complication by measuring the sternal and ribs thickness preoperatively using chest CT scan that helped in accurately choice the proper size screw. Using the drill guide and an accurately measured screw length with the depth gauge intraoperatively at time played an important role in minimizing post-operative bleeding in our series. We only have one case (2.5%) of post-op bleeding and that was not related to plates or screws. The bleeder was found in the subcutaneous tissue that was surgically ligated. Cicilioni [19] reported two post-op bleeding (4%) in his series. Both bleeding events were from an intercostals and a pectoral vessel and thus was not plate-related. They were easily recognized and treated. Violation of the pleural space can occur during the debridement, drilling or during the insertion, and this occurred in one of our patients. Huh [20] had no incidence of pneumothorax or injury to the underlying heart or vascular structures.

In addition to restoration of sternal stability following sternal plating, prevention of post-operative infection requires excellent debridement, lavage irrigation with saline and aggressive postoperative antibiotic coverage. We have made it our practice to aggressively excise all wound edges, including skin, subcutaneous tissue, any necrotic -appearing tissue including chronic granulation tissue present down to the level of the sternal bone. The goal of debridement is to convert the chronic wound into an acute one. All nonviable bone and cartilage were removed down to a level that bleeds. Subsequently, the entire wound was vigorously lavaged with 3 liters of warm normal saline. We had only 2 patients developed postoperative wound infection. The first patient had end stage renal failure with chronic hemodialysis, uncontrolled IDDM and PVD that required bilateral leg amputation. During his hospital course, he developed sternal wound infection that initially required VAC therapy and subsequently underwent sternal fixation. He required hardware removal. At the time of his hardware removal, the sternal bone was found to be well healed. The soft tissue wound was successfully managed with VAC therapy. He was treated with a second course of IV antibiotics and went on to complete healing. The second patient was on chronic steroid use because of long standing chronic asthma. His wound responded well to wound treatment and antibiotics. Cicilioni et al., [19] encountered only a 2.7% incidence of recurrent infection and suggest that titanium plates have a bacteriostatic property.

We achieved thoracic stability in all our patients. Using traditional strategies, such as parasternal Robicsek weave with or without muscle flap, Olbrecht [15], noticed that 20% of his patients had post-operative sternal dehiscence.

We did not remove any plates because of loosening although others had had this complication [19,21]. One author Huh et al., [20] had to remove the plates in two patients (14%) associated with infection in one and plate fracture in another. Plate fracture leading to instability is a concern. Repeated bending weaken the strength of a plate at one point may be a factor.

Patients complain of chronic pain following surgery for treatment of sternal dehiscence and sternal osteomyelitis. We only have two patients (5%) who had chronic post-operative pain. The first patient responded well to medical management and the second one required plate removal to relieve his pain.

However, one author [22] removed 50% of the transverse placed plates, due to persisting pain. Another [23] reported that 51% of their patients complained of chronic chest or shoulder pain following sternal wound reconstruction using muscle or omental flaps.

### Limitations of the study

The number of the patients in this series is relatively small group of the 1200 cases undergoing open heart surgery at our institution every year. However, this approach to sternal infection/dehiscence has changed our practice. Presently, sternectomy is rarely performed and more extensive soft tissue reconstruction techniques are not required. Preservation of the anatomical and functional aspects of the sternum is possible. However, the physiologic effect of this approach in preserving the sternum and the effect of the plating itself, require further study. In addition, being a retrospective review study in one centre, the benefit and integration of this approach into present practice requires further study. A larger multi-center prospective control trials using larger number of patients, comparing different techniques of sternal reconstruction are needed to address the indications, benefits and potential complications of this approach.

## Conclusions

Sternal plating appears to be an effective option for the treatment of sternal dehiscence as it yields a stable sternum. It is simple and safe technique without risks. Long-term follow-up and further larger studies are needed to address the indications, benefits and complications of sternal plating.

## Competing interests

The authors declare that they have no competing interests.

## Authors' contributions

HF conceived of the study, participated in its design, carried out the operations, collecting and analyzing the data, writing, reviewing and submitting the manuscript. KO participated in collecting and analyzing the data. LE participated in reviewing the manuscript. DL participated in reviewing the manuscript. DB participated in reviewing the manuscript. MM participated in performing the operations and reviewing the manuscript. JM conceived of the study, participated in its design, carried out the operations, writing and reviewing the manuscript. All authors read and approved the final manuscript.
